# Adaptation of a plant pathogen to partial host resistance: selection for greater aggressiveness in grapevine downy mildew

**DOI:** 10.1111/eva.12368

**Published:** 2016-02-24

**Authors:** Chloé E. L. Delmas, Frédéric Fabre, Jérôme Jolivet, Isabelle D. Mazet, Sylvie Richart Cervera, Laurent Delière, François Delmotte

**Affiliations:** ^1^ UMR 1065 Santé et Agroécologie du VignobleINRAVillenave d'OrnonFrance; ^2^ Bordeaux Science AgroUMR 1065 SAVEISVVUniversité de BordeauxVillenave d'OrnonFrance

**Keywords:** erosion, evolvability, fitness cost, host specificity, obligate plant pathogen, quantitative resistance, virulence, *Vitis vinifera*

## Abstract

An understanding of the evolution of pathogen quantitative traits in response to host selective pressures is essential for the development of durable management strategies for resistant crops. However, we still lack experimental data on the effects of partial host resistance on multiple phenotypic traits (aggressiveness) and evolutionary strategies in pathogens. We performed a cross‐inoculation experiment with four grapevine hosts and 103 isolates of grapevine downy mildew (*Plasmopara viticola*) sampled from susceptible and partially resistant grapevine varieties. We analysed the neutral and adaptive genetic differentiation of five quantitative traits relating to pathogen transmission. Isolates from resistant hosts were more aggressive than isolates from susceptible hosts, as they had a shorter latency period and higher levels of spore production. This pattern of adaptation contrasted with the lack of neutral genetic differentiation, providing evidence for directional selection. No specificity for a particular host variety was detected. Adapted isolates had traits that were advantageous on all resistant varieties. There was no fitness cost associated with this genetic adaptation, but several trade‐offs between pathogen traits were observed. These results should improve the accuracy of prediction of fitness trajectories for this biotrophic pathogen, an essential element for the modelling of durable deployment strategies for resistant varieties.

## Introduction

The use of quantitative resistance (i.e. partial resistance) to control pathogens is increasingly being seen as a valuable and durable approach to crop protection (Brun et al. 2010; Lê Van et al. 2013; Mundt 2014; Niks et al. 2015). However, quantitative disease resistance is also considered to be a prime driver of the quantitative evolution of host–pathogen interactions, and there is some concern that it may select for pathogen aggressiveness, also referred to as quantitative pathogenicity (Grenfell et al. 2004; Lannou 2012; Burdon et al. 2014; Caffier et al. 2014; Mundt 2014). Despite the applied importance of studying the quantitative evolution of pathogen life‐history traits with host selective pressures, we still lack experimental data on the effects of partial host resistance on multiple phenotypic traits (aggressiveness) and evolutionary strategies in pathogens.

The existence of phenotypic variation in aggressiveness is a key factor necessary for pathogen adaptation (Sacristan and Garcia‐Arenal 2008; Lannou 2012; Zhan and McDonald 2013). Aggressiveness can be assessed by evaluating multiple phenotypic quantitative traits of the pathogen directly linked to its fitness. These traits are likely to be under selection, resulting in differential adaptive patterns according to the environment (Pariaud et al. 2009; Lannou 2012). The host exerts selective pressures on the quantitative traits of the pathogen, and its ability to resist pathogens is particularly important in this respect. Gandon and Michalakis (2000) showed, in a theoretical study, that host quantitative resistance selected for greater virulence (i.e. the amount of damage caused to the host) in parasites, assuming that quantitative resistance had no direct effect on pathogen transmission. This model predicted that once quantitative plant resistance is eroded, pathogens exhibited greater virulence not only on the resistant host, but also on fully susceptible hosts. Similarly, Gandon et al. (2001) and Read et al. (2015) showed, theoretically and experimentally, respectively, that the evolution of pathogens in response to imperfect vaccines (reducing pathogen growth rate like quantitative resistance) resulted in higher levels of intrinsic virulence. These predictions cannot be directly transposed to plant–pathogen systems, as quantitative resistance in the plant often affects pathogen transmission (Lannou 2012). Further, properly evaluating the adaptive potential of pathogens to host variety required the estimation of phenotypic and genetic trait variance (Lynch and Walsh 1998). In practice, it implies to sample a large number of isolates on clearly identified hosts and measure, in cross‐inoculation experiments, quantitative traits for each isolate individually. Previous experimental studies on fungal pathogens and oomycetes have yet suggested that the erosion of partial host resistance comes with the selection of greater pathogen aggressiveness (reviewed in Pariaud et al. 2009 and Mundt 2014). However, these studies were not designed to disentangle the relative role of phenotypic and genetic trait variance in pathogen adaptation required to estimate short‐term evolutionary potential (Hansen et al. 2011).

The potential of pathogens to evolve in response to host selective pressures is constrained by trade‐offs between quantitative traits (Laine and Barrès 2013; Susi and Laine 2013). Indeed, basic evolutionary theory involves trade‐offs between fitness components, in the form of negative relationships between two quantitative traits, each of which is positively correlated with fitness (Stearns 1992). The adaptation of pathogens to particular abiotic environments or host genotypes thus results from a complex pattern of coevolved quantitative traits defining the evolutionary strategy of pathogens in these conditions. Several trade‐offs between the quantitative traits of plant pathogens (Lannou 2012) and viruses (Froissart et al. 2010) have been reported, including trade‐offs between transmission and latency period duration (Héraudet et al. 2008; Pariaud et al. 2013) and between transmission and virulence, defined as the amount of damage caused to the host (Doumayrou et al. 2013).

The potential of pathogens to evolve in response to host selective pressures may also be constrained by fitness costs associated with adaptation to partial resistance (‘cost of aggressiveness’). For example, a recent study of experimental evolution in potato virus Y showed that adaptation to quantitative resistance was associated with a fitness cost on the susceptible cultivar (Montarry et al. 2012). Fitness costs associated with quantitative adaptation of the pathogen have received much less attention (Zhan and McDonald 2013) than the fitness costs associated with the ability to overcome qualitative host resistance (Leach et al. 2001; Thrall and Burdon 2003; Sacristan and Garcia‐Arenal 2008; Bahri et al. 2009; Montarry et al. 2010). A full understanding of the potential of pathogens to evolve when faced with partial host resistance will require further studies on a large panel of host–isolate interactions in controlled conditions, and assessments both of trade‐offs between multiple quantitative pathogenicity traits and of the strength of counter selection on susceptible hosts. Such studies will constitute a key step towards the design of effective strategies for managing plant pathogens and preserving the durability of partial host resistance.

We investigated the selective effects of partial host resistance on the quantitative phenotypic traits of *Plasmopara viticola*, the agent of grapevine downy mildew. Downy mildew is a highly destructive disease of grapevines in all vine‐growing areas of the world (Galet 1977; Gessler et al. 2011) and it is recognized as one of the most damaging oomycete pathogens on the basis of socio‐economic criteria (Kamoun et al. 2015). This plant pathosystem is ideal for studies of quantitative trait evolution in response to partial resistance, because partially resistant grapevine varieties have recently been deployed over a small geographical scale in Europe. This situation provides us with a unique opportunity to monitor the evolutionary response of *P. viticola* populations faced with this new selective pressure. Recent evidence from studies of two commercial resistant varieties suggests that *P. viticola* can rapidly erode partial resistance even in conditions of limited deployment in vineyards (Peressotti et al. 2010; Casagrande et al. 2011; Delmotte et al. 2014). We carried out a large‐scale sampling of *P. viticola* isolates from partially resistant grapevine varieties and susceptible *V. vinifera* cultivars (‘RES’ and ‘SUS’ isolate origins, respectively). We performed a controlled cross‐inoculation experiment and analysed phenotypic differentiation between the two isolate origins and four grapevine hosts inoculated, for five quantitative traits. The genetic differentiation of ‘RES’ and ‘SUS’ isolates was assessed with 32 microsatellite markers.

We thus investigated the genetic adaptation of *P. viticola* to grapevine partial resistance, correlations between quantitative traits, pathogen specificity to resistant varieties and fitness costs associated with adaptation, addressing four questions: Is there adaptive (phenotypic) and/or neutral genetic differentiation between pathogen populations collected from partially resistant and susceptible hosts? Which quantitative traits are involved in adaptation to host resistance and how are they correlated? Do *P. viticola* isolates adapt specifically to their hosts of origin or is there a global adaptation to partial resistance? Is there a fitness cost associated with adaptation to partial resistance?

## Materials and methods

### Study system

Grapevine, a perennial plant, was originally domesticated from a unique Eurasian species in the Caucasian area (*Vitis vinifera* L.; Zecca et al. 2012), and modern varieties are cultivated worldwide. Since the middle of the 19th century, viticulture has been threatened by grapevine downy mildew, which was introduced into Europe from North America (Millardet 1881). *Vitis vinifera* cultivars are highly susceptible to grapevine downy mildew, which is caused by *Plasmopara viticola*, an obligate biotrophic oomycete (Viennot‐Bourgin 1949). This pathogen completes its life cycle on the host plant by asexual reproduction, causing lesions on leaves and clusters, and sexual reproduction to produce the overwintering oospores. Genetic resistance has been introgressed into *V. vinifera* from wild American and Asian *Vitis spp*. and conventional breeding programmes for resistance to fungal pathogens have recently resulted in the creation of commercial varieties with partial resistance to *P. viticola* (Bouquet et al. 2000; Spring 2003; Eibach et al. 2007; Burger et al. 2009; ICV 2013). A few partially resistant varieties have been deployed over very small areas in Europe. The Regent variety, for example, was first deployed 17 years ago and now covers 1.94% of the total vineyard area in Germany (*Vitis* International Variety Catalogue; www.vivc.de).

### Pathogen and host materials

In 2012, *P. viticola* isolates were collected from the border area between France, Switzerland and Germany, where partially resistant grapevines are grown (Figure S1). Samples were collected from 22 vineyards (Table 1 and Table S1; Figure S1) in five vine‐growing regions (Alsace–Baden, Burgundy, Tessino, Vaud–Valais and Zurich). Each isolate consisted of a single sporulating lesion from an infected leaf (oil spot). Isolates were stored in liquid nitrogen for further use. We collected samples from two types of host: susceptible cultivars of *V. vinifera* (hereafter referred to as the ‘SUS’ isolate origin) and partially resistant varieties (hereafter referred to as the ‘RES’ isolate origin). We collected 103 isolates in total: 49 from nine susceptible *V. vinifera* cultivars (‘SUS’ isolate origin) and 54 from 13 partially resistant varieties (‘RES’ isolate origin) (Table S1). ‘RES’ and ‘SUS’ isolates were never collected from the same vineyard.

**Table 1:** Number of *Plasmopara viticola* isolates collected per vine‐growing region from susceptible cultivars (‘SUS’) and partially resistant varieties (‘RES’)

Vine‐growing region	Number of locations	‘SUS’	‘RES’
Alsace–Baden	6	18	26
Burgundy	3	8	0
Tessino	2	0	11
Vaud–Valais	7	14	10
Zurich	4	9	7
Total	22	49	54

Four host plants were used for the inoculation experiment: the susceptible host *V. vinifera* cv. Cabernet sauvignon and three partially resistant grapevine hosts: Regent, Bronner and Prior. These resistant varieties were released onto the market in 1995, 1999 and 2008, respectively. They had therefore been cultivated for a maximum of 17, 13 and 4 years, respectively, at the time of pathogen sampling in 2012. Little is known about the determinism of the genetic resistance in these commercial varieties, but some studies have highlighted resistance factors in Regent and Bronner. Three QTL have been identified in Regent: Rpv3, Rpv4 and Rpv11 (Fisher et al. 2003; Merdinoglu et al. 2003; Welter et al. 2007; Bellin et al. 2009; Moreira et al. 2011; Schwander et al. 2012). The principal QTL underlying resistance in Bronner is Rpv10 (Blasi 2010; Schwander et al. 2012). Nothing is known about the resistance of Prior, but this variety has ancestors in common with Bronner and these two varieties may therefore share some resistance factors. Plants were grafted onto the SO4 rootstock and grown simultaneously in a glasshouse under natural photoperiod conditions. Full pedigrees for partially resistant varieties and *V. vinifera* cultivars can be found in the *Vitis* International Variety Catalogue (www.vivc.de).

### Controlled cross‐inoculation experiment

We generated 103 inocula for this experiment, by propagating each isolate of *P. viticola* on detached leaves from glasshouse‐grown *V. vinifera* cv. Cabernet sauvignon plants in Petri dishes stored in controlled conditions (20°C, 12‐h/12‐h day/night photoperiod). On the day before the cross‐inoculation experiment, we gently washed the sporulating leaves to remove the sporangia. Leaves were placed in growth chambers for 1 day to ensure the production of sporangia of the same age for all isolates. The inoculum for each isolate was obtained by collecting sporangia from leaves in sterile water. Its concentration was adjusted to 10 000 sporangia/mL with a portable particle counter (Scepter 2.0^™^ automated cell counter; Millipore, Darmstadt, Germany).

We prepared plant leaf discs for inoculation, by collecting the third and fourth leaves below the apex of young shoots from each cultivar at the ten‐unfolded‐leaf stage. Leaves were rinsed with distilled water and leaf discs of 15 mm in diameter were excised with a cork borer. We used five replicate leaf discs for the susceptible inoculated host, and three for each of the three resistant inoculated hosts. We therefore inoculated 1545 leaf discs in total, to study 412 plant–pathogen interactions (103 isolates × 4 host plants). For each isolate‐inoculated host interaction, leaf discs were floated on the surface of the inoculum, adaxial side up, for 4 h at 20°C. Inoculated leaf discs were randomized and placed abaxial side up on damp filter paper in square Petri dishes (23 × 23 cm). The Petri dishes were sealed with cling film once the discs dried and were placed for 5 days in a phytotron (Conviron CMP 5090; Winnipeg, MB, Canada) with a 12‐h/12‐h light/dark photoperiod, at 20°C.

### Molecular characterization

For each isolate, we retained one inoculated leaf disc (cv. Cabernet sauvignon) for DNA extraction after sporangia collection (see quantitative trait description below). Leaf discs were freeze‐dried overnight and DNA was extracted by the standard cetyl‐trimethyl‐ammonium bromide–phenol–chloroform method with precipitation in isopropanol. DNA was resuspended in sterile water and the volume was then made up to 150 μL. We amplified 32 microsatellite loci by multiplex PCR: Pv7, Pv14, Pv16, Pv17, Pv31, Pv39 (Delmotte et al. 2006); Pv101, Pv91, Pv103, Pv138, Pv143, Pv147, Pv65, Pv93, Pv104, Pv135, Pv137, Pv141, Pv148, Pv76, Pv83, Pv87, Pv88, Pv126, Pv139, Pv61, Pv127, Pv134, Pv142, Pv146, Pv124 (Rouxel et al. 2012); and ISA (Gobbin et al. 2003). PCR was performed in a volume of 6 μL containing 2.5 μL of H_2_O 1.5 μL of QIAGEN Multiplex Master Mix (QIAGEN, Hilden, Germany), 0.5 μL of primer mix and 1.5 μL of DNA. The PCR programme, performed in an Eppendorf Mastercycler EP gradient (Eppendorf, Montesson, France), consisted of initial denaturation at 95°C for 15 min and 35 cycles of 30 s at 94°C 1 min at 55°C, and 45 s at 72°C, ending with a 30 s extension at 60°C. PCR products (1–1.5 μL diluted 1:14) were mixed with 10 μL of formamide and 0.14 μL of internal lane size standard (GeneScan 600 LIZ; Thermo Fisher Scientific, Waltham, MA, USA) and analysed in an ABI 3130 capillary sequencer according to the manufacturer's instructions (Applied Biosystems, Thermo Fisher Scientific). DNA fragments were automatically sized with GeneMapper^™^ v4.0 software (Applied Biosystems, Thermo Fisher Scientific).

### Genetic structure analysis

Genetic diversity (number of alleles, mean number of alleles per locus and global expected heterozygosity) was assessed with GENETIX v4.05.2 (Belkhir et al. 2004)**.** Multilocus genotypes were identified with GENODIVE software, using a stepwise mutation model (Meirmans and Van Tienderen 2004). Population differentiation (*F*_ST_) was estimated for pathogen origin (‘RES’ versus ‘SUS’), and for vine region of origin (five regions; Figure S1). The 95% confidence interval of the *F*_ST_ was generated with GENETIX, by bootstrap analysis of the original data with 1000 replicates. We investigated genetic structure, by performing principal component analysis (PCA) with the adegenet package in R (Jombart 2008). Multivariate analysis is particularly suitable for plant–pathogen species that are partially clonal, because this method is hypothesis‐free, and therefore does not require Hardy–Weinberg equilibrium, for example (Dutech et al. 2010).

### Pathogen aggressiveness

We first quantified three elementary traits of the pathogen life cycle: sporangium production (hereafter called ‘spore production’), sporangium size (hereafter called ‘spore size’) and latency period (Table 2). Spore production and spore size were assessed 5 days postinoculation (dpi), with a Multisizer 3 automatic particle counter (Coulter Counter^®^ Multisizer^™^ 3; Beckman Coulter, Brea, CA, USA). We gently washed each leaf disc separately in 10 mL of saline (Isoton II; Beckman Coulter) to collect sporangia. We determined the number of sporangia per mm² (cumulative, over 5 days of infection) and weighted sporangium size, as described by Delmas et al. (2014). The latency period was estimated by visually checking leaf discs daily under a stereomicroscope and recording the day of first sporulation for each disc. The latency period was defined as the time interval between inoculation and the first record of sporangia.

**Table 2:** List of quantitative pathogenicity traits in *Plasmopara viticola*

Quantitative trait	Definition	Method	Unit
**Main pathogenicity traits**			
Spore production	Final number of sporangia per mm² of leaf disc (5 dpi)	Particle analyser	Sporangia/mm^2^
Spore size	Weighted average sporangium size	Particle analyser	μm
Latency period	Time interval between inoculation and the onset of sporulation	Visual estimation under a stereomicroscope	dpi
**Sporulation dynamics**			
*T*_50_	Time to 50% of final sporulation (5 dpi) based on sporulation dynamics over time	Image analysis, curve fitting	d
Sporulation rate	Slope at *T*_50_	Image analysis, curve fitting	Percentage of leaf disc area displaying sporulation/d

d, days; dpi, days postinoculation.

We also estimated the sporulation dynamics of each infected leaf disc, using two traits, time to 50% of final sporulation (*T*_50_) and sporulation rate (slope at *T*_50_; Table 2). The estimation of these traits was independent of the estimation of final sporulation level. *T*_50_ and sporulation rate are model parameters estimated from the dynamics of pathogen infection monitored over time by image analysis. Each day, from 1 to 5 dpi, we took pictures of leaf discs with a camera (Canon EOS 650D) equipped with a macro lens (Canon EF 100 mm f/2.8 USM) installed on a camera copy stand (Kaiser Statif RS1). Images were analysed with a simple semi‐automatic method in Image J software (Peressotti et al. 2011). This method quantifies sporulation as pixels contrasting with the leaf disc in the background. Sporulation is expressed as the proportion of the leaf disc area that is sporulating. We fitted a logistic model to these experimental data (Fig. 2) with PROC NLIN in SAS Studio (SAS University Edition; version 3.3; SAS Institute Inc., Cary, NC, USA) as follows:

$$Y_{ixr}=\frac{1}{1+{\left(T_{50}/x\right)}^{\mathrm{slope}}}+\varepsilon_{ixr}$$

where the observed variables are *x* (time, from 0 to 5 dpi) and *Y*_*irx*_ (the relative sporulating leaf disc area which is the proportion of the leaf area displaying sporulation of isolate *i* repetition *r* on day *x* divided by the proportion of the leaf area displaying sporulation of isolate *i* repetition *r* on day 5). *Y*_*ird*_ ranged from 0 to 1 and was independent of spore production 5 dpi.

### Quantitative trait analysis

We investigated whether quantitative pathogenicity traits differed between inoculated hosts (phenotypic plasticity) and pathogen origins (genetic adaptation), by performing a mixed general model analysis for each quantitative trait with PROC GLIMMIX in SAS software (SAS University Edition). We used the following model: *Y*_*ijk*_ = *μ *+ *H*_*i*_ + *P*_*j*_ + *H*_*i*_ *P*_*j*_ + *I(P)*_*k*_ **+ *ɛ*_*ijk*_ where *H* is the inoculated host *i* (fixed factor), *P* is the pathogen origin *j* (fixed factor), *HP* their interaction (fixed factor) and *I(P)* is the isolate *k* nested in the pathogen origin (random factor). The error term of this model, *ɛ*, therefore corresponds to within‐isolate variation (i.e. differences between replicates). We compared quantitative traits between pathogen origins (‘RES’ versus ‘SUS’) on each inoculated host, by determining *post hoc* least squares means of fixed effects.

For all models, we plotted studentized marginal and conditional residuals, to check that residuals are independent and identically, and normally distributed. For three of the five traits measured (spore production, latency period and the slope at *T*_50_), log transformation of the data was required to satisfy these requirements. Finally, we checked for spatial autocorrelation in model residuals using Moran's I parametric test implemented in the PROC VARIOGRAM in SAS Software. Spatial autocorrelation was not detected in any of the models (see Table 3).

**Table 3:** Effects of ‘inoculated host’ (Bronner, Prior, Regent and *Vitis vinifera* cv. Cabernet sauvignon) and ‘pathogen origin’ (‘RES’ and ‘SUS’) on quantitative pathogenicity traits in *Plasmopara viticola,* as defined in Table 2. Type III statistics of fixed effects from mixed linear models are presented. ‘Inoculated host’ and ‘pathogen origin’ were fixed effects and the isolate was nested in ‘pathogen origin’ and entered into the model as a random effect. Statistically significant results (*P* < 0.05) are shown in bold. See the text for the model specificity for each trait. A Moran's I parametric test was performed on model residuals: no significant spatial autocorrelation was detected in any traits (*Z*‐scores and *P*‐values are reported)

Source	Inoculated host (*H*)	Pathogen origin (*P*)	Interaction (*H* × *P*)	Moran's I
**Main pathogenicity traits**				
Spore production	*F*_3,1287_ = 753.80	*F*_1,101_ = 21.97	*F*_3,1287_ = 12.51	−0.419
	***P *** **<** *** *** **0.0001**	***P *** **<** *** *** **0.0001**	***P *** **<** *** *** **0.0001**	*P *=* *0.68
Spore size	*F*_3,1287_ = 308.16	*F*_1,101_ = 14.24	*F*_3,1287_ = 4.88	−1.397
	***P *** **<** *** *** **0.0001**	***P *** **=** *** *** **0.0003**	***P *** **=** *** *** **0.0022**	*P *=* *0.16
Latency period	*F*_3,1303_ = 185.35	*F*_1,101_ = 28.89	*F*_3,1303_ = 23.73	−1.24
	***P *** **<** *** *** **0.0001**	***P *** **<** *** *** **0.0001**	***P *** **<** *** *** **0.001**	*P *=* *0.21
**Sporulation dynamics**				
*T*_50_	*F*_3,1243_ = 228.07	*F*_1,101_ = 3.13	*F*_3,1243_ = 25.99	−1.290
	***P *** **<** *** *** **0.0001**	*P *=* *0.08	***P *** **<** *** *** **0.0001**	*P *=* *0.20
Sporulation rate	*F*_3,1243_ = 160.54	*F*_1,101_ = 2.33	*F*_3,1243_ = 1.94	−0.93
	***P *** **<** *** *** **0.0001**	*P *=* *0.13	*P *=* *0.12	*P *=* *0.35

Overall trait correlations were explored with Pearson correlation statistics (PROC CORR), by the application of Fisher's *Z* transformation to data averaged for each interaction (isolate × inoculated host).

### Host specificity

We checked for specificity for the resistant host variety in the isolates collected from partially resistant varieties (‘RES’), by testing the local versus foreign hypothesis. On each inoculated resistant host *j,* we compared aggressiveness of ‘local’ (pathogen isolates collected from a focal resistant host and used to inoculate the same resistant host) and ‘foreign’ (pathogen isolates collected from a focal resistant host and used to inoculate a different resistant host) isolates in a mixed general model (PROC GLIMMIX), as follows: *Y*_*k*_ = *μ *+ *LF* + *I(LF)*_*k*_ **+ *ɛ*_*k*_ where *LF* is the local versus foreign effect on host *j* (fixed factor) and *I(LF)* is the isolate *k* nested in the LF contrast (random factor). The local isolates used were collected from Regent and used to inoculate Regent (*n *=* *7), collected from Bronner/Solaris and used to inoculate Bronner (*n *=* *9) and collected from Prior and used to inoculate Prior (*n *=* *10). The isolates collected from Bronner and Solaris were pooled together, as these two resistant varieties are full siblings (*Vitis* International Variety Catalogue; www.vivc.de). On each inoculated host, foreign isolates included all isolates collected from a resistant variety (‘RES’) other than the local isolates (*n *=* *46 used to inoculate Regent; *n *=* *37 used to inoculate Bronner and *n *=* *44 used to inoculate Prior). As described above, a log transformation of the data was carried out for three of the five traits assessed (spore production, latency period and the slope at *T*_50_) to ensure that the requirements for normality and independence of residuals were satisfied.

For more detailed analyses of host specificity, we further compared the quantitative pathogenicity traits of isolates by source host Bronner, Prior, Regent, all other partially resistant varieties and susceptible *V. vinifera* cultivars. This analysis is presented in Method S2, Table S2 and Figure S2.

## Results

### Genetic structure analysis

**Figure 1:**
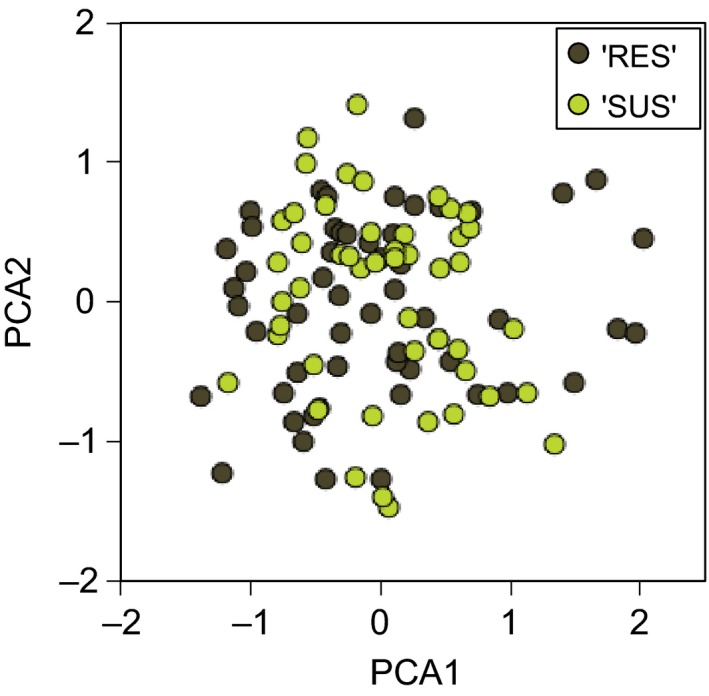
Principal component analysis (PCA) on multilocus genotypes based on 32 microsatellite markers, for the 103 *Plasmopara viticola* isolates. Pathogen origins are indicated in colour on the PCA. ‘SUS’: 49 isolates collected from the susceptible host (pale green); ‘RES’: 54 isolates collected from partially resistant hosts (dark green).

Overall, 88 alleles were detected at the 32 microsatellite loci, with two to six alleles per locus. The mean number of alleles per locus was 3.66, and expected heterozygosity reached 0.376 ± 0.19. We identified 103 different multilocus genotypes, indicating that all isolates were genetically different. Axes 1 and 2 of the PCA accounted for 9.2% and 7.3% of total genetic variability, respectively. PCA analysis did not group isolates by pathogen origin (‘RES’ or ‘SUS’) (Fig. 1). No significant genetic differentiation was observed between pathogen origin (*F*_ST_ = −0.0012, 95% CI: [−0.0046: 0.0020]; *P *=* *0.10) and vine‐growing region of origin (*F*_ST_ = −0.008, 95% CI: [−0.0115: 0.0214], *P *=* *0.4).

### Factors affecting pathogen aggressiveness

Brown tanning was observed on 133 of the 1545 leaf discs inoculated, indicating a premature degeneration of plant tissue and precluding the reliable assessment of pathogen sporulation. We therefore excluded these discs from the analysis, resulting in a final sample size of 1412 leaf discs and 401 plant–pathogen interactions (on Bronner *n *=* *46 ‘RES’ and *n *=* *47 ‘SUS’ isolates; on Regent *n *=* *53 ‘RES’ and *n *=* *49 ‘SUS’ and on Prior and *V. vinifera* cv. Cabernet sauvignon *n *=* *54 ‘RES’ and *n *=* *49 ‘SUS’). All leaf discs but 13 presented sporulation 5 dpi (12 from the Bronner variety and 1 from Cabernet sauvignon cv.). The final level of sporulation recorded on the susceptible host Cabernet sauvignon reached, on average, 640 ± 20 sporangia/mm^2^ (Table S3; Fig. 3), of the same order of magnitude as previous reports for this pathosystem (Delmas et al. 2014; Delmotte et al. 2014).

Overall, we found significant effects of inoculated host on all quantitative traits and of pathogen origin (genetic adaptation) on three of the five traits tested: spore production, spore size and latency period (Table 3). The interaction of these two effects (G × E) was significant for all quantitative traits other than sporulation rate, indicating a dependence of the effect of inoculated host (resistance efficacy) on pathogen origin (‘RES’ origin versus ‘SUS’ origin).

The significantly lower levels of spore production on the three resistant hosts than on the susceptible host provided an indication of the overall efficacy of disease resistance (Fig. 3A; Table S3). On resistant varieties, spore production levels were lowest on Bronner (92% less spore production than on the susceptible host), intermediate on Prior (71%) and highest on Regent (spore production levels 58% lower than on the susceptible host). Bronner and Prior also had the longest latency periods (Fig. 3C; Table S3). [Correction added on 26 March 2016 after initial online publication: The figure citation in the above sentence was changed to ‘Fig. 3C’ from ‘Fig. 3B’.].

### Pathogen adaptation to partial host resistance

**Figure 2:**
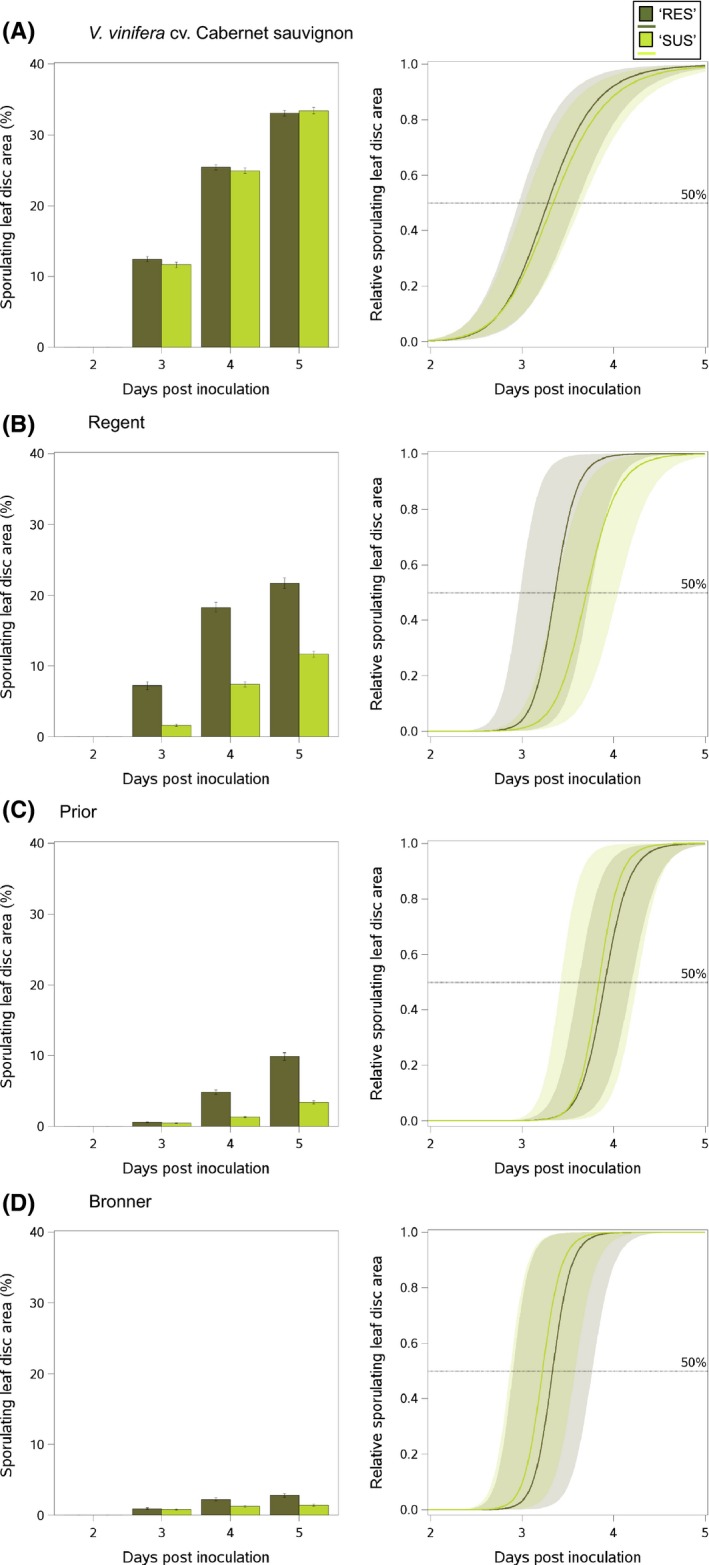
Sporulation dynamics of *Plasmopara viticola* isolates from partially resistant hosts (‘RES’; dark green; *n *=* *54) and susceptible host (‘SUS’; light green; *n *=* *49). Isolates were used to inoculate (A) *Vitis vinifera* cv. Cabernet sauvignon, (B) Regent, (C) Prior and (D) Bronner. On the left‐hand side of the figure, the mean percentage (±SE) of the leaf disc area displaying sporulation (experimental data obtained from image analysis) is presented from 0 to 5 days post‐inoculation (dpi). On the right‐hand side of the figure, sporulation curves resulting from the adjustment of a logistic model (mean ± 95% confidence intervals) for the experimental data are presented for each pathogen origin (‘SUS’ and ‘RES’). The relative sporulating leaf disc area presented on the *y*‐axis is the proportion of the leaf disc displaying sporulation on day *x* divided by the sporulating area on day 5. Horizontal lines correspond to 50% of final sporulation (5 dpi). From these curves, we estimated *T*_50_ as the time to reach the 50% and sporulation rate, and the slope at *T*_50_ (Table 2). See the text for details of the logistic model and Table 3 for statistical differences between inoculated hosts and pathogen origins.

**Figure 3:**
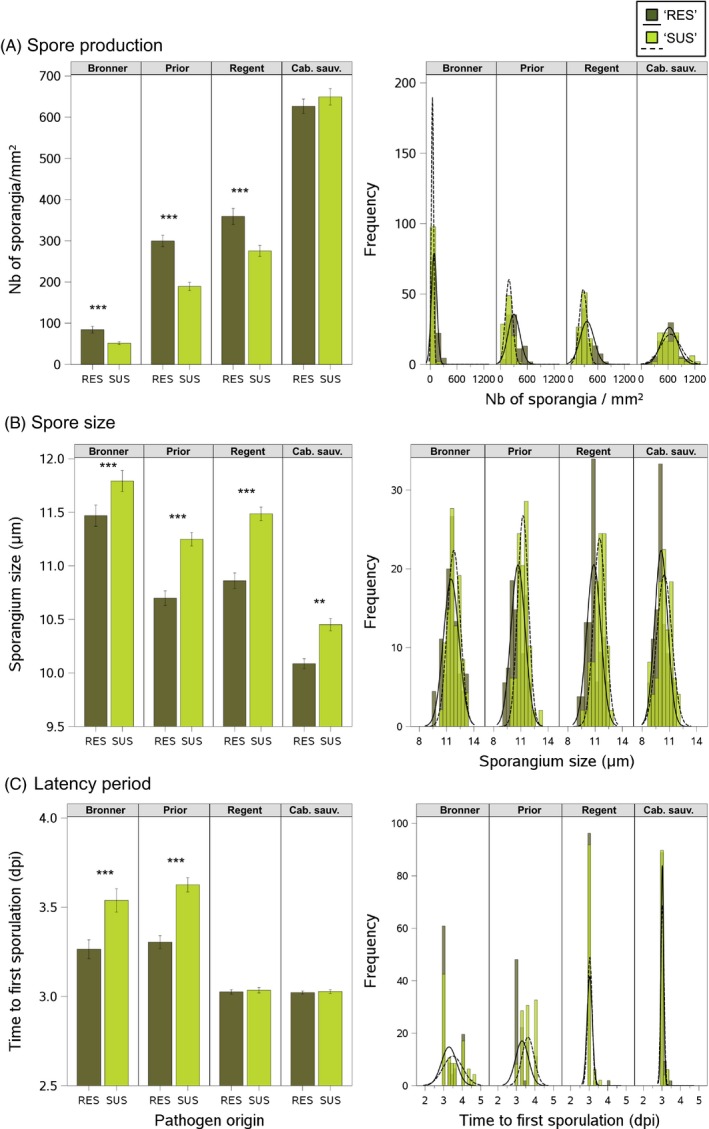
Quantitative pathogenicity traits of *Plasmopara viticola* isolates. The pathogen origin ‘RES’ includes isolates collected from resistant varieties (dark green; solid line; *n *=* *54) and the pathogen origin ‘SUS’ includes isolates collected from susceptible *Vitis vinifera* cultivars (light green; dashed line; *n *=* *49). Isolates were used to inoculate Bronner, Prior, Regent and *V. vinifera* cv. Cabernet sauvignon (Cab. sauv.). For each quantitative trait (A: spore production; B: spore size and C: latency period), mean ± SE values of isolates by pathogen origin and frequency density plots are presented for each inoculated host on the left‐ and right‐hand side panels, respectively. See Table 3 for full model statistics. *Post hoc* least squares mean differences between pathogen origins (‘SUS’ and ‘RES’) on each inoculated host were determined and the significance of these differences is indicated as follows: ***P <* *0.0001; **P <* *0.001; *P <* *0.05.

We found significant differences in spore production and spore size between ‘SUS’ and ‘RES’ isolate host origins on the three partially resistant hosts (Fig. 3A,B). Isolates of ‘RES’ origin produced significantly larger numbers of spores than those of ‘SUS’ origin on Regent (+31%), Prior (+59%) and Bronner (+72%) (Table S3). The spores of isolates of ‘RES’ origin were significantly smaller than those of ‘SUS’ isolates on Regent (−5.6%), Prior (−4.9%) and Bronner (−2.5%) (Table S3). Other traits differed significantly between host origins on one (*T*_50_ and sporulation rate) or two (latency period) inoculated hosts. Isolates of ‘RES’ origin had a significantly shorter latency period (Fig. 3C; Table S3) on Bronner (−5.81%) and Prior (−8.8%) and a significantly lower *T*_50_ (−9.6%, indicating faster sporulation dynamics) and higher sporulation rate (+33%) on Regent than isolates of ‘SUS’ origin (Fig. 2; Table S3).

Distribution analyses of all the traits studied demonstrated a shift in the mean distribution of isolates of ‘RES’ origin associated with a higher variance, but the distributions of isolates of the two origins still overlapped (Fig. 3 right panels; Figure S3).

### Quantitative trait correlations

**Figure 4:**
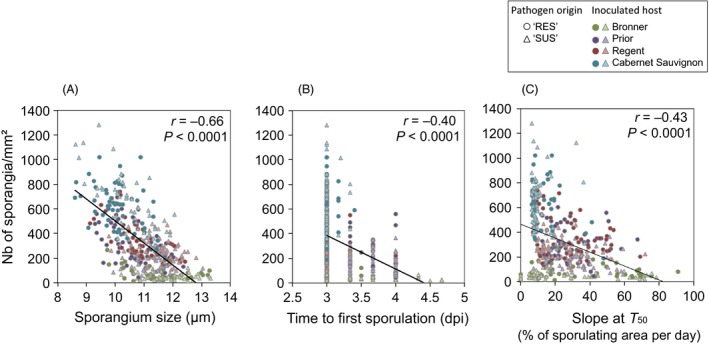
Quantitative trait correlations for 103 *Plasmopara viticola* isolates collected from susceptible grapevine cultivars (‘SUS’, triangles; *n *=* *49) and partially resistant grapevine varieties (‘RES’, circles, *n *=* *54). Spore production decreased with (A) increasing spore size, (B) increasing latency period duration and (C) increasing sporulation rate. Isolates were used to inoculate Bronner (green), Prior (purple), Regent (red) and *Vitis vinifera* cv. Cabernet sauvignon (blue). The overall correlation is indicated with a black solid line. Full statistics for all correlations tested are presented in Table 4.

We found significant overall correlations for eight of 10 pairs of traits (Table 4). Spore production decreased significantly with increasing spore size (Fig. 4A), increasing latency period (Fig. 4B) and increasing sporulation rate (Fig. 4C). Accordingly, spore size was significantly and positively correlated with latency period and sporulation rate (Table 4). Latency period increased significantly with increasing *T*_50_ and sporulation rate (Table 4) and *T*_50_ decreased significantly with increasing sporulation rate (Table 4).

**Table 4:** Pearson correlation analysis (Fisher *Z* transformation) for five quantitative pathogenicity traits of *Plasmopara viticola* isolates sampled on two different types of hosts (susceptible grapevine cultivars and partially resistant varieties) and used to inoculate four different grapevine hosts. Statistically significant results (*P* < 0.05) are shown in bold. Sample size *N* indicates the number of interactions (isolates × inoculated hosts)

Variable	Covariable	*N*	*r* (Fisher *Z*)	*P*‐value
Spore production	Spore size	400	**−0.79680**	**<0.0001**
Spore production	Latency period	400	**−0.42388**	**<0.0001**
Spore production	*T*_50_	394	−0.01083	0.8304
Spore production	Sporulation rate	394	**−0.47192**	**<0.0001**
Spore size	Latency period	400	**0.26545**	**<0.0001**
Spore size	*T*_50_	394	−0.07126	0.1588
Spore size	Sporulation rate	394	**0.29294**	**<0.0001**
Latency period	*T*_50_	394	**0.13494**	**0.0076**
Latency period	Sporulation rate	394	**0.44907**	**<0.0001**
*T*_50_	Sporulation rate	394	**−0.16057**	**0.0015**

### Host specificity

**Figure 5:**
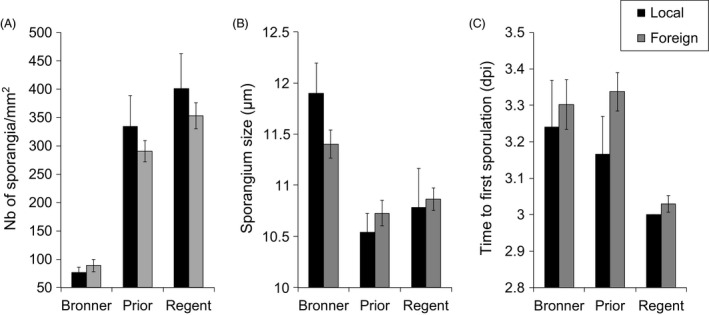
Specificity of *Plasmopara viticola* isolates for resistant grapevine varieties. None of the differences between local and foreign isolates was significant (main pathogenicity traits), on any of the inoculated hosts (Table 5). Mean (±SD) values for quantitative pathogenicity traits (A: spore production; B: spore size; C: latency period) are presented for each inoculated resistant host, for local and foreign isolates. Local isolates were collected from a focal resistant variety and used to inoculate the same variety (Bronner: *n *=* *9; Prior: *n *=* *10; Regent: *n *=* *7), whereas foreign isolates were collected from a focal resistant variety and used to inoculate a different variety (Bronner: *n *=* *37; Prior: *n *=* *44; Regent: *n *=* *46).

We found no evidence of host specificity for the resistant varieties concerning the main pathogenicity traits (spore production, spore size and latency period). ‘Local’ pathogen isolates did not differ significantly from ‘foreign’ isolates for any of these quantitative traits, on any of the three inoculated resistant hosts (Fig. 5; Table 5), demonstrating a lack of specificity for resistant host variety. However, we found little evidence of specificity for the resistant variety when considering traits related to sporulation dynamics. The sporulation rate of local isolates was lower than that of foreign isolates on Prior (*P *=* *0.04; Table 5; Figure S4). *T*_50_ tend to be smaller for local than for foreign isolates on Regent and tend to be greater for local than foreign isolates on Bonner (*P *=* *0.07 and *P *=* *0.08, respectively; Table 5; Figure S4).

**Table 5:** Effects of ‘local versus foreign’ pathogen origin on quantitative pathogenicity traits in *Plasmopara viticola,* for each resistant host inoculated (Bronner, Prior, Regent). Differences in least squares means (LSM) between local isolates and foreign isolates were determined with mixed linear models. The local isolates used were those collected from Bronner (*n *=* *9), Prior (*n *=* *10) and Regent (*n *=* *7) and tested on the Bronner, Prior and Regent hosts, respectively. Foreign isolates included all other isolates collected from a resistant variety (*n *=* *37 for Bronner, *n *=* *46 for Regent and *n *=* *44 for Prior). The isolate nested in the ‘local versus foreign’ effect was entered into the model as a random effect. Statistically significant results (*P* < 0.05) are shown in bold

Host inoculated	Trait	LSM	SE	DF	*t*‐Value	*P*‐value
**Main pathogenicity traits**						
Bronner	Spore production	0.012	0.113	43	0.107	0.915
	Spore size	0.494	0.313	43	1.579	0.122
	Latency period	−0.007	0.019	44	−0.377	0.708
Prior	Spore production	0.027	0.083	52	0.328	0.744
	Spore size	−0.186	0.274	52	−0.680	0.500
	Latency period	−0.021	0.015	52	−1.397	0.168
Regent	Spore production	0.093	0.077	51	1.208	0.233
	Spore size	−0.079	0.319	51	−0.248	0.805
	Latency period	−0.004	0.007	51	−0.493	0.624
**Sporulation dynamics**						
Bronner	*T*_50_	0.231	0.128	41	1.800	0.079
	Sporulation rate	−0.124	0.132	41	−0.935	0.355
Prior	*T*_50_	−0.014	0.067	52	−0.202	0.841
	Sporulation rate	−0.124	0.059	52	−2.108	**0.040**
Regent	*T*_50_	−0.238	0.129	51	−1.843	0.071
	Sporulation rate	0.064	0.110	51	0.584	0.562

### Fitness cost associated with adaptation

We found no apparent differences in quantitative traits between isolates of ‘RES’ origin and of ‘SUS’ origin on the susceptible host, suggesting that there was no fitness cost associated with aggressiveness. Indeed, spore production, latency period and *T*_50_ on the susceptible host did not differ between isolates of ‘SUS’ and ‘RES’ origin (Figs 2 and 3A,C; Figure S3). By contrast, isolates of ‘RES’ origin had smaller spores (*P *=* *0.01; Fig. 3B) and tended to have higher sporulation rates (*P *=* *0.03; Fig. 2; Figure S3). Moreover, the distributions of all the quantitative traits on the susceptible host overlapped fully between isolates of ‘RES’ and ‘SUS’ origins (Fig. 3 and Figure S3 right panels). [Correction added on 26 March 2016 after initial online publication: The figure citation of figure 3 was changed for the below occurrences: (i) In the first paragraph of left column the citation was changed to ‘Fig. 3A,B’ from ‘Fig. 3A– D’ and ‘Fig. 3C’ from ‘Fig. 3E,F’ (ii) In the above paragraph to ‘Fig. 3A,C’ from ‘Fig. 3A,E’ and ‘Fig. 3B’ from ‘Fig. 3C’.].

## Discussion

### Partial host resistance selects for greater aggressiveness

Despite the considerable interest in the use of partially resistant plants for controlling crop pathogens, little is known about the evolutionary response of multiple quantitative traits in plant pathogens to the selective pressure exerted by such plant resistance (Lannou 2012, but see Caffier et al. 2014). We found that pathogen isolates collected from partially resistant hosts (‘RES’) were significantly more aggressive (i.e. greater quantitative pathogenicity) than the ‘naïve’ isolates collected from susceptible hosts (‘SUS’). This genetic adaptation was observed regardless of the resistant host inoculated and involved the main pathogenicity traits of *P. viticola*. The adapted isolates produced significantly larger numbers of smaller spores (sporangia) than the ‘naïve’ isolates. The adapted isolates had shorter latency periods associated with a shorter time to 50% final sporulation (*T*_50_) and a higher sporulation rate. From an epidemiological perspective, these features may confer several advantages on these isolates, including a greater capacity for transmission to healthy resistant hosts. In addition, a shorter latency period, shorter *T*_50_ and higher sporulation rate may render the pathogen more competitive with other pathogens, as shorter latency has been shown to lead to better performance in the field (Antonovics and Alexander 1989; Pringle and Taylor 2002). Increases in the aggressiveness of adapted isolates may have important consequences for epidemics and asexual propagation of the pathogen on partially resistant hosts (Iacono et al. 2012). Despite the erosion of resistance resulting from the adaptation of *P. viticola* isolates, it is important to keep in mind that our results also indicated that grapevine varieties still efficiently controlled the pathogen by significantly decreasing its sporulation on leaves.

### Quantitative trait correlations

Selection for greater pathogen aggressiveness could theoretically be hampered by trade‐offs between pathogen life‐cycle traits (Anderson and May 1982; Frank 1996; Koella and Agnew 1999; Paul et al. 2004). We found that spore production was negatively correlated with three quantitative traits: spore size, latency period and sporulation rate. The negative correlation between sporangium number and size found for these 103 pathogen isolates confirms previous findings published by Delmas et al. (2014) and Delmotte et al. (2014). In *P. viticola*, sporangium production (and therefore transmission capacity) is positively correlated with zoospore production (infection capacity), regardless of sporangium size (Delmas et al. 2014). Adapted isolates of *P. viticola* producing more sporangia are thus more infective in the next generation of the pathogen cycle and probably have a selective advantage, even if they produce smaller sporangia. In addition, high levels of spore production were correlated with a short latency period, which would confer a competitiveness advantage on aggressive isolates. A similar phenotypic correlation has been found between latency period and transmission success in the plant pathogen *Hyaloperonospora arabidopsidis* (Héraudet et al. 2008) and between latency period and spore production capacity in the wheat leaf rust pathogen, *Puccinia triticina* (Pariaud et al. 2013). However, high levels of spore production were correlated with slow sporulation, potentially counteracting the benefits of a shorter latency period. Trait correlations have been shown to differ between inoculations in sympatry and in allopatry (Susi and Laine 2013), suggesting that life‐history trade‐offs may influence epidemiological dynamics. We found that the strength of the correlations observed depended on the interaction considered (inoculated host × pathogen isolate as shown in Fig. 4), indicating similar mechanisms in *P. viticola*. The consequences of these genetic trait correlations on epidemiological dynamics remained to be investigated.

### Quantitative trait differentiation

The impact of host selective pressures on pathogen quantitative traits could theoretically result in a shift in the distribution of the traits (different ranges of trait values for the adapted and naïve isolates, scenario 1) or an adaptive peak corresponding to a portion of the phenotypic diversity of the naïve population (shift in trait average, scenario 2). These scenarios depend on host and pathogen genetic backgrounds and their interactions (Lannou 2012). In this study, the shift in the mean values observed, with many values still overlapping between the distributions in adapted and naïve isolates of *P. viticola,* was suggestive of the second of these evolutionary scenarios. Moreover, adaptation to partial resistance was accompanied by an increase in trait coefficient of variations, particularly for spore production and spore size (Table S3). As resistant grapevine varieties are sparsely distributed in a landscape of susceptible *V. vinifera* cultivars, ‘RES’ isolates are probably a mixture of adapted isolates (that have undergone several cycles on the resistant variety) and nonadapted isolates, due to potential continuous pathogen migration from the surrounding susceptible *V. vinifera* cv. This process probably underlies the higher variability of traits and suggests that we may underestimate the strength of selection.

Furthermore, comparisons of the divergence of quantitative traits and neutral molecular markers in populations can suggest which evolutionary processes underly population differentiation for multiple quantitative traits: genetic drift, directional selection or uniform selection (McKay and Latta 2002; reviewed in Leinonen et al. 2013). We compared two groups of isolates (adapted versus naïve) and found no evidence of neutral genetic differentiation at 32 microsatellite markers. European populations of *P. viticola* have already been shown to present a weak continent‐wide genetic structure and low levels of genetic diversity, consistent with the occurrence of a bottleneck at the time of introduction in the 1870s (Fontaine et al. 2013). The lack of population genetic structure observed at this spatial scale contrasts with the significant trait differences between pathogen origins on each resistant host inoculated. Quantitative trait divergence apparently largely exceeded neutral expectations, suggesting a pattern of adaptation probably driven by directional selection resulting in different phenotypic optima in naïve and adapted *P. viticola* populations (Merilä and Crnokrak 2001).

In addition, we can estimate here the short‐term evolutionary potential of spore production as spore production was shown to be highly different between adapted (‘RES’) and naïve (‘SUS’) pathogen origins on each resistant host inoculated. The evolutionary potential can be approximate as the expected proportional change in spore production under a unit strength of selection, that is evolvability (Houle 1992; Hansen et al. 2011). We therefore estimated the additive genetic variance of this trait and calculated its evolvability (mean‐scaled additive genetic variance; see Method S1 and Table S4) (Houle 1992; Hansen and Houle 2008; Hansen et al. 2011; Garcia‐Gonzalez et al. 2012). According to Hansen et al. (2011), the per cent change in spore production in *t* generations given an evolvability of *e*_*μ*_ and a mean‐scaled directional selection gradient of *β*_*μ*_ is approximated by (1 + *e*_*μ*_ *β*_*μ*_)^*t*^ (Method S1). Setting *β*_*μ*_ to 0.3 (its median value from the meta‐analysis of Hereford et al. (2004)), and *e*_*μ*_ to its value estimated in the present study (Table S4), we were able to estimate the number of generation necessary to reach the divergence observed among adapted (‘RES’) and naïve (‘SUS’) pathogen origins. We obtained 8 generations in Bronner, 9 generations in Prior and 20 generations in Regent (Table S4). Remarkably, these estimates are close to the time elapsed since the release of each resistant variety (around 17 years for Regent, 13 years for Prior, 4 years for Bronner). This crude calculation suggests that the divergence among isolates is compatible with a pattern of usual directional selection (Hereford et al. 2004), over the time scale corresponding to the age of populations. Each year, grapevine downy mildew is realizing a single generation of sexual reproduction to overwinter and several cycles of asexual reproduction during the cropping season (Gessler et al. 2011). Thus, the short‐term dynamics of downy mildew adaptation to resistant varieties is likely mainly driven by this single annual event of sexual reproduction.

### Weak (if any) evidence of host specificity for resistant varieties

Many pathogens display some degree of host specialization (Ploch et al. 2010; Thines et al. 2010; Choi et al. 2011; Telle et al. 2011; Rouxel et al. 2013). This is likely due to the heterogeneity of selection pressures across hosts (Giraud et al. 2010), to selection mosaics (Nuismer 2006) and to the relative gene flow rates of host versus pathogen species (Hoeksema and Forde 2008). It has also been proposed that pathogen maladaptation is relatively uncommon when host–pathogen interactions are governed by quantitative traits (Ridenhour and Nuismer 2007). In agro‐ecosystems, populations of rapidly evolving fungal pathogen species may quantitatively adapt to the most common cultivars encountered in the landscape (Bonman et al. 1989; Enjalbert et al. 2005; Montarry et al. 2006; Andrivon et al. 2007). We determined whether local isolates collected from a partially resistant host in the field were more aggressive than foreign isolates on the resistant variety from which they originated. *P. viticola* adaptation, with the development of greater aggressiveness, was found to be global and nonspecific, because quantitative adaptation to a given host was found to be similar for pathogen populations tested on their host of origin and for foreign pathogens. We only found marginally significant evidence of host specificity when looking at the sporulation dynamic parameters. This result is supported by the diversity of source hosts sampled in our experiment, as the ‘RES’ pathogen isolates came from 13 different partially resistant varieties. Aggressiveness therefore appears to be conferred by several mutations, such that pathogens selected on a partially resistant host possess quantitative traits that are advantageous on other partially resistant varieties. Thus, although the disease resistance of the varieties tested in our controlled cross‐inoculation was conferred by different genetic factors (see the materials and methods), the mechanisms underlying the resistance may not be sufficiently different to result in specific adaptation to grapevine varieties.

### No apparent fitness cost associated with adaptation

The costs associated with pathogen adaptation to hosts are among the most important factors determining the rate and extent of adapted isolate emergence (Aubertot et al. 2006; Pietravalle et al. 2006). Fitness costs have classically been considered in studies of the gene‐for‐gene plant–pathogen interaction (‘cost of virulence’; see Van Der Plank 1963), but have rarely been addressed in the context of partial resistance. We investigated possible fitness costs on the susceptible host *V. vinifera* cv. Cabernet sauvignon by comparing quantitative pathogenicity traits (as a proxy for fitness) between adapted and naïve *P. viticola* isolates. We detected no differences between adapted and naïve pathogens on *V. vinifera* for three of the quantitative traits assessed (spore production, *T*_50_ and latency period). However, on the susceptible host, adapted isolates produced smaller spores and tended to have higher sporulation rates, suggesting a lower infection potential in the next generation (Delmas et al. 2014) and faster sporulation dynamics on susceptible hosts, respectively. However, the lack of evidence for fitness costs should be interpreted with care. We cannot exclude the possibility of fitness costs detectable only in competitive situations (co‐infections) or for traits relating to the sexual part of the life cycle of *P. viticola*, as these two fitness components were not assessed here. Moreover, this study was performed in optimal conditions for pathogen development, which may have decreased the chances of detecting small differences in quantitative traits on the highly susceptible Cabernet sauvignon cultivar. Finally, fitness costs may have been counteracted by compensatory mutations. Indeed, second‐site compensatory mutations have been shown to reduce the deleterious effect of another mutation on fitness (Kimura 1990). This situation has been widely reported for antimicrobial resistance, in which cost compensation seems to be the rule rather than the exception in a broad range of organisms (Björkman et al. 2000; Levin et al. 2000; Maisnier‐Patin and Andersson 2004).

### Consequences for the durability of partially resistant varieties

Models explicitly linking within‐host population genetics and evolutionary processes and between‐host epidemiological processes are needed to achieve sustainable plant resistance management. Over recent decades most theoretical studies have focused on the management of qualitative resistance gene (Mundt 2014), with recently a particular interest for management strategies designed at landscape scales (e.g. Fabre et al. 2015). Few theoretical studies deal with the management of quantitative resistance impacting differentially several life‐history traits of the pathogen (but see Iacono et al. 2012; Bourget et al. 2015). This required certainly to model the fitness trajectories of pathogen populations over time on both susceptible and resistant hosts (Lannou 2012; Mundt 2014) but also to collect extensive data set. To this respect, the results reported here for *P. viticola* are the first quantitative data to be published concerning the assessment of fitness‐related traits on a range of varieties. We found that the partially resistant varieties still effectively controlled the pathogen (mean rates of spore production 74% lower than those on susceptible cv. Cabernet sauvignon), but that their resistance had been rapidly eroded due to the adaptation of pathogen genotypes. Molecular markers indicated that the adaptation of *P. viticola* to the different partially resistant varieties was not due to the emergence and spread of a unique clonal genotype with greater aggressiveness as reported in other plant pathogenic oomycetes (Ahmed et al. 2012; Cooke et al. 2012). The resistant varieties tested here have only been on the market for a few years in Europe: 17 years for Regent, 13 years for Prior, 4 years for Bronner. All have been planted over very limited geographic areas. For example, in Germany, the country in which such varieties have been most widely used, Regent is the commonest variety used, but it still accounts for only 1.94% of the total vineyard area (http://www.vivc.de; *Vitis International Variety Catalogue*). Despite this recent and limited deployment of resistance, *P. viticola* populations have rapidly responded to this selective pressure, consistent with the high evolutionary potential of *P. viticola* already reported for fungicide resistance (Chen et al. 2007; Blum et al. 2010). Rapidly evolving pathogens greatly complicate the task of preserving the durability of plant resistance genes (McDonald and Linde 2002). This is particularly true for *P. viticola*, in which we found no host variety specificity and no apparent fitness cost associated with this global adaptation.

This study demonstrated the rapid and general erosion of partial resistance in grapevine downy mildew. From a theoretical point of view, it raises important questions about the genetic architecture underlying the evolution of quantitative pathogenicity traits. Few studies have addressed this issue, and the genetic determinism of pathogen aggressiveness remains poorly understood (Lannou 2012). New insight into the genomic architecture of quantitative traits can be gained from QTL approaches based on the analysis of progenies, genomewide association studies comparing natural populations or experimental evolution studies coupled to resequencing of the pathogen genome (Lambrechts 2010; Guidot et al. 2014). From an applied point of view, our results suggest that the deployment of resistant varieties should be associated with other control methods (biological control, sanitation, fungicide) if we are to achieve sustainable management of grapevine disease.

## Data archiving statement

Data available from the Dryad Digital Repository: http://dx.doi.org/10.5061/dryad.b59g7.

## Supporting information


**Method S1.** Evolvability of spore production in different resistant grapevine varieties.
**Method S2.** Cross‐inoculation analysis.
**Table S1.** Details of the *Plasmopara viticola* isolates used in the study.
**Table S2.** Mixed linear models of the effects of inoculated host and source host (Bronner, Prior, Regent, other partially resistant varieties, *V. vinifera* cultivars) and their interaction on quantitative pathogenicity traits in *P. viticola*.
**Table S3.** Quantitative traits of *P. viticola* isolates from two origins (‘RES’ isolates collected from partially resistant grapevine hosts and ‘SUS’ isolates collected from susceptible *V. vinifera* cultivars) and used to inoculate Bronner, Prior, Regent and *V. vinifera* cv. Cabernet sauvignon.
**Table S4.** Evolvability and additive genetic variance of spore production in *P. viticola* isolates from different origins, for each inoculated resistant variety.
**Figure S1.** Map of *Plasmopara viticola* sampling, at 22 locations within five vine‐growing regions: Burgundy, Alsace–Baden, Vaud–Valais, Tessino and Zurich.
**Figure S2.** Specificity of *P. viticola* isolates for their source host of origin.
**Figure S3.** Quantitative traits characterizing the sporulation dynamics of *P. viticola* isolates from two origins (‘RES’ isolates collected from partially resistant grapevine hosts and ‘SUS’ isolates collected from susceptible *V. vinifera* cultivars).
**Figure S4.** Specificity of *P. viticola* isolates for resistant grapevine varieties considering traits related to sporulation dynamics.?
