# Supplementary material for: Adaptation of a plant pathogen to partial host resistance: selection for greater aggressiveness in grapevine downy mildew
Source: Evol Appl. 2016 Feb 24;9(5):709–25. doi: 10.1111/eva.12368 (PMC4869412; doi:10.1111/eva.12368)
Supplement: Supplementary file 1 — Method S1. Evolvability of spore production in different resistant grapevine varieties. Method S2. Cross‐inoculation analysis. Table S1. Details of the Plasmopara viticola isolates used in the study. Table S2. Mixed linear models of the effects of inoculated host and source host (Bronner, Prior, Regent, other partially resistant varieties, V. vinifera cultivars) and their interaction on quantitative pathogenicity traits in P. viticola. Table S3. Quantitative traits of P. viticola isolates from two origins (‘RES’ isolates collected from partially resistant grapevine hosts and ‘SUS’ isolates collected from susceptible V. vinifera cultivars) and used to inoculate Bronner, Prior, Regent and V. vinifera cv. Cabernet sauvignon. Table S4. Evolvability and additive genetic variance of spore production in P. viticola isolates from different origins, for each inoculated resistant variety. Figure S1. Map of Plasmopara viticola sampling, at 22 locations within five vine‐growing regions: Burgundy, Alsace–Baden, Vaud–Valais, Tessino and Zurich. Figure S2. Specificity of P. viticola isolates for their source host of origin. Figure S3. Quantitative traits characterizing the sporulation dynamics of P. viticola isolates from two origins (‘RES’ isolates collected from partially resistant grapevine hosts and ‘SUS’ isolates collected from susceptible V. vinifera cultivars). Figure S4. Specificity of P. viticola isolates for resistant grapevine varieties considering traits related to sporulation dynamics.? [file EVA-9-709-s001.docx]

## Supporting Information

Additional Supporting Information may be found in the online version of this article:

**Description**

**Method S1.** Evolvability of spore production in different resistant grapevine varieties

**Method S2.** Cross-inoculation analysis

**Table S1.** Details of the *Plasmopara viticola* isolates used in the study

**Table S2.** Mixed linear models of the effects of inoculated host and source host (Bronner, Prior, Regent, other partially resistant varieties, *V. vinifera* cultivars) and their interaction on quantitative pathogenicity traits in *P. viticola*

**Table S3.** Quantitative traits of *P. viticola* isolates from two origins (‘RES’ isolates collected from partially resistant grapevine hosts and ‘SUS’ isolates collected from susceptible *V. vinifera* cultivars) and used to inoculate Bronner, Prior, Regent and *V. vinifera* cv. Cabernet sauvignon

**Table S4.** Evolvability and additive genetic variance of spore production in *P. viticola* isolates from different origins, for each inoculated resistant variety.

**Figure S1.** Map of *Plasmopara viticola* sampling, at 22 locations within five vine-growing regions: Burgundy, Alsace-Baden, Vaud-Valais, Tessino and Zurich

**Figure S2.** Specificity of *P. viticola* isolates for their source host of origin.

**Figure S3.** Quantitative traits characterizing the sporulation dynamics of *P. viticola* isolates from two origins

**Figure S4**. Specificity of *P. viticola* isolates for resistant grapevine varieties considering traits related to sporulation dynamics

**Method S1.** *Evolvability of spore production*. For each inoculated resistant host, we used the following model: *Y*_jk_ = µ + *P_j_* + *I(P)_k_* + *ɛ_jk_* where *P* is the pathogen origin *j* (fixed factor) and *I(P)* is the isolate *k* nested in the pathogen origin (random factor). The error term of this model, *ɛ*, therefore corresponds to within-isolate variation (i.e. differences between replicates). We obtained the additive genetic variance (V_A_) and its standard error (genetic variance between isolates nested within a origin, i.e. ‘SUS or ‘RES’) using a mixed general model analysis for each quantitative trait with PROC GLIMMIX in SAS software (SAS University Edition). In cross-inoculation experiments with asexually reproducing species such as *P. viticola*, any variance among replicates can theoretically be attributed to environmental effects because different replicates of an isolate represent the same genotype (i.e. they are identical clones) (Zhan et al. 2005).

We calculated the mean-scaled evolvability as *e_μ_* = V_A_/m^2^, where m is the overall mean in spore production (SUS and RES pooled) (Hansen and Houle 2008; Hansen et al. 2011). We calculated the observed percent change (absolute value) among SUS and RES origins (spore production; SPO) for each resistant inoculated host as following: (SPO_SUS_ – SPO_RES_)/SPO_RES_ x 100.

Finally, the percent change over *t* generations with an evolvability of *e_μ_* and strength of selection *β_μ_* can be approximate to (1 + *e_μ_β_μ_*)^t^ according to Hansen et al. (2011). To estimate the number of generation (*t*) necessary to obtained the observed percent change among pathogen origins (SUS *vs*. RES) on the resistant hosts, we used β_μ_ = 0.3, the median bias-corrected estimate of multivariate gradients from the meta-analysis of Hereford et al. (2004).

**References (Methods S1)**

Hansen, T. F., and D. Houle 2008. Measuring and comparing evolvability and constraint in multivariate characters. Journal of Evolutionary Biology **21**:1201-1219.

Hansen, T. F., C. Pelabon and D. Houle 2011. Heritability is not Evolvability. Evolutionary Biology. **38**:258-277.

Hereford, J., T. F. Hansen, and D. Houle 2004. Comparing strengths of directional selection: How strong is strong? Evolution **58** :2133–2143.

Zhan J., C. C., Linde, T., Jurgens, U., Merz, F., Steinebrunner and B.A., McDonald 2005. Variation for neutral markers is correlated with variation for quantitative traits in the plant pathogenic fungus *Mycosphaerella graminicola*. Molecular Ecology **14**:2683–2693.

**Method S2.** *Cross-inoculation analysis.* For more detailed analyses of host specificity, we further compared the quantitative pathogenicity traits of isolates by source host (1) Bronner/Solaris (*n* = 10 isolates), (2) Prior (*n* = 10), (3) Regent (*n* = 8 isolates) and (4) collected from the other partially resistant varieties (*n* = 26 isolates) and from (5) the susceptible *V. vinifera* cultivars (*n* = 49 isolates).

We used a mixed general model (PROC GLIMMIX, SAS) as follows: *Y*_ijk_ = µ + *H_i_* + *S_j_* + *H_i_S_j_* + *I(S)_k_* + *ɛ_ijk_* where *H* is the inoculated host *i* (fixed factor), *S* is the source host of origin *j* (fixed factor), *HS* their interaction (fixed factor), *I(S)* is the isolate *k* nested in the source host *S* (random factor). We plotted Studentized marginal and conditional residuals, to check the normality, identity and independence of the residuals of each trait. For four of the five traits measured (spore production, latency period and the slope at *T*_50_), log-transformation of the data was required to satisfy these requirements.

**Table S1.** Details of the *Plasmopara viticola* isolates used in the study. Isolates were collected in 2012 from the border area between France, Switzerland and Germany, where partially resistant grapevines are grown. Samples were collected from 22 vineyards in five vine-growing regions (Alsace-Baden, Burgundy, Tessino, Vaud-Valais and Zurich). Each isolate consisted of a single sporulating lesion from an infected leaf (oil spot). Isolates were collected from two types of host: ’SUS’, susceptible cultivars of *V. vinifera* (*n* = 9 cultivars) and ‘RES’, partially resistant varieties (*n* = 13 partially resistant genotypes). Full pedigree for partially resistant varieties and *V. vinifera* cultivars can be found in the *Vitis* International Variety Catalogue ([www.vivc.de](http://www.vivc.de)).

| Isolate | Vine region | x_long | y_lat | Site  number | Pathogen origin | Source host  genotype |
| --- | --- | --- | --- | --- | --- | --- |
| PV1760 | Alsace-Baden | 7.331336 | 48.063138 | 1 | RES | Bianca |
| PV1762 | Alsace-Baden | 7.331336 | 48.063138 | 1 | RES | Bianca |
| PV1764 | Alsace-Baden | 7.331336 | 48.063138 | 1 | RES | Bianca |
| PV1528 | Alsace-Baden | 7.331336 | 48.063138 | 1 | RES | Johanniter |
| PV1325 | Alsace-Baden | 7.331336 | 48.063138 | 1 | RES | Regent |
| PV1332 | Alsace-Baden | 7.331336 | 48.063138 | 1 | RES | Regent |
| PV1519 | Alsace-Baden | 7.331336 | 48.063138 | 1 | RES | Villard blanc |
| PV1369 | Alsace-Baden | 7.62282 | 48.05266 | 2 | RES | Baron |
| PV1376 | Alsace-Baden | 7.62282 | 48.05266 | 2 | RES | Johanniter |
| PV1381 | Alsace-Baden | 7.62282 | 48.05266 | 2 | RES | Johanniter |
| PV1186 | Alsace-Baden | 7.62282 | 48.05266 | 2 | SUS | Pinot blanc |
| PV1189 | Alsace-Baden | 7.62282 | 48.05266 | 2 | SUS | Pinot blanc |
| PV1271 | Alsace-Baden | 7.62282 | 48.05266 | 2 | SUS | Pinot blanc |
| PV1170 | Alsace-Baden | 7.62282 | 48.05266 | 2 | SUS | Pinot noir |
| PV1174 | Alsace-Baden | 7.62282 | 48.05266 | 2 | SUS | Pinot noir |
| PV1508 | Alsace-Baden | 7.62282 | 48.05266 | 2 | RES | Regent |
| PV1747 | Alsace-Baden | 7.62282 | 48.05266 | 2 | RES | Solaris |
| PV1750 | Alsace-Baden | 7.62282 | 48.05266 | 2 | RES | Solaris |
| PV1306 | Alsace-Baden | 7.77644 | 47.95964 | 3 | SUS | Gewurztraminer |
| PV1312 | Alsace-Baden | 7.77644 | 47.95964 | 3 | SUS | Gewurztraminer |
| PV1480 | Alsace-Baden | 7.77644 | 47.95964 | 3 | RES | Johanniter |
| PV1481 | Alsace-Baden | 7.77644 | 47.95964 | 3 | RES | Johanniter |
| PV1211 | Alsace-Baden | 7.76351 | 47.9358 | 4 | SUS | Chasselas |
| PV1263 | Alsace-Baden | 7.76351 | 47.9358 | 4 | RES | Johanniter |
| PV1514 | Alsace-Baden | 7.76351 | 47.9358 | 4 | RES | Johanniter |
| PV1438 | Alsace-Baden | 7.76351 | 47.9358 | 4 | RES | Prior |
| PV1443 | Alsace-Baden | 7.76351 | 47.9358 | 4 | RES | Prior |
| PV1444 | Alsace-Baden | 7.76351 | 47.9358 | 4 | RES | Prior |
| PV1445 | Alsace-Baden | 7.76351 | 47.9358 | 4 | RES | Prior |
| PV1348 | Alsace-Baden | 7.76351 | 47.9358 | 4 | RES | Regent |
| PV1492 | Alsace-Baden | 7.76351 | 47.9358 | 4 | RES | Regent |
| PV1301 | Alsace-Baden | 7.73332 | 47.92876 | 5 | SUS | Chasselas |
| PV1318 | Alsace-Baden | 7.73332 | 47.92876 | 5 | SUS | Chasselas |
| PV1252 | Alsace-Baden | 7.73332 | 47.92876 | 5 | SUS | Pinot noir |
| PV1255 | Alsace-Baden | 7.73332 | 47.92876 | 5 | SUS | Pinot noir |
| PV1280 | Alsace-Baden | 7.73332 | 47.92876 | 5 | SUS | Pinot noir |
| PV1282 | Alsace-Baden | 7.73332 | 47.92876 | 5 | SUS | Pinot noir |
| PV1740 | Alsace-Baden | 7.73332 | 47.92876 | 5 | RES | Solaris |
| PV1741 | Alsace-Baden | 7.73332 | 47.92876 | 5 | RES | Solaris |
| PV1742 | Alsace-Baden | 7.73332 | 47.92876 | 5 | RES | Solaris |
| PV1473 | Alsace-Baden | 7.22785 | 47.9095 | 6 | SUS | Gewurztraminer |
| PV1476 | Alsace-Baden | 7.22785 | 47.9095 | 6 | SUS | Gewurztraminer |
| PV1478 | Alsace-Baden | 7.22785 | 47.9095 | 6 | SUS | Gewurztraminer |
| PV1166 | Alsace-Baden | 7.22785 | 47.9095 | 6 | SUS | Pinot gris |
| PV1622 | Zurich | 8.677389 | 47.224972 | 7 | RES | Baron |
| PV1809 | Zurich | 8.677389 | 47.224972 | 7 | RES | Baron |
| PV1660 | Zurich | 8.677389 | 47.224972 | 7 | RES | Cabernet carbon |
| PV1663 | Zurich | 8.677389 | 47.224972 | 7 | RES | Cabernet carbon |
| PV1706 | Zurich | 8.677389 | 47.224972 | 7 | RES | Regent |
| PV1692 | Zurich | 8.677389 | 47.224972 | 7 | RES | Solaris |
| PV1773 | Zurich | 8.677389 | 47.224972 | 7 | RES | Solaris |
| PV1641 | Zurich | 8.76786 | 47.23457 | 8 | SUS | *V. vinifera* |
| PV1645 | Zurich | 8.76786 | 47.23457 | 8 | SUS | *V. vinifera* |
| PV1651 | Zurich | 8.77616 | 47.23855 | 9 | SUS | *V. vinifera* |
| PV1654 | Zurich | 8.77616 | 47.23855 | 9 | SUS | *V. vinifera* |
| PV1768 | Zurich | 8.77616 | 47.23855 | 9 | SUS | *V. vinifera* |
| PV1142 | Zurich | 8.813243 | 47.226827 | 10 | SUS | *V. vinifera* |
| PV1146 | Zurich | 8.813243 | 47.226827 | 10 | SUS | *V. vinifera* |
| PV1668 | Zurich | 8.813243 | 47.226827 | 10 | SUS | *V. vinifera* |
| PV1671 | Zurich | 8.813243 | 47.226827 | 10 | SUS | *V. vinifera* |
| PV1948 | Tessino | 8.948336 | 46.178761 | 11 | RES | Isabella |
| PV1952 | Tessino | 8.948336 | 46.178761 | 11 | RES | Isabella |
| PV1900 | Tessino | 8.916378 | 46.187912 | 12 | RES | Leon Millot |
| PV1902 | Tessino | 8.916378 | 46.187912 | 12 | RES | Leon Millot |
| PV1413 | Tessino | 8.916378 | 46.187912 | 12 | RES | Monarch |
| PV1383 | Tessino | 8.916378 | 46.187912 | 12 | RES | Prior |
| PV1384 | Tessino | 8.916378 | 46.187912 | 12 | RES | Prior |
| PV1385 | Tessino | 8.916378 | 46.187912 | 12 | RES | Prior |
| PV1878 | Tessino | 8.916378 | 46.187912 | 12 | RES | Prior |
| PV1881 | Tessino | 8.916378 | 46.187912 | 12 | RES | Prior |
| PV1884 | Tessino | 8.916378 | 46.187912 | 12 | RES | Prior |
| PV1638 | Vaud-Valais | 7.372403 | 46.240747 | 13 | SUS | *V. vinifera* |
| PV1639 | Vaud-Valais | 7.372403 | 46.240747 | 13 | SUS | *V. vinifera* |
| PV1589 | Vaud-Valais | 7.218736 | 46.183857 | 14 | RES | Cabernet Cortis |
| PV1935 | Vaud-Valais | 7.218736 | 46.183857 | 14 | RES | Cabernet Cortis |
| PV1119 | Vaud-Valais | 7.218736 | 46.183857 | 14 | SUS | Cornalin |
| PV1122 | Vaud-Valais | 7.218736 | 46.183857 | 14 | SUS | Cornalin |
| PV1125 | Vaud-Valais | 7.218736 | 46.183857 | 14 | SUS | Cornalin |
| PV1136 | Vaud-Valais | 7.218736 | 46.183857 | 14 | SUS | V. vinifera |
| PV1131 | Vaud-Valais | 7.218736 | 46.183857 | 14 | SUS | *V. vinifera* (blanc) |
| PV1602 | Vaud-Valais | 7.218736 | 46.183857 | 14 | SUS | *V. vinifera* (blanc) |
| PV1606 | Vaud-Valais | 7.218736 | 46.183857 | 14 | SUS | *V. vinifera* (blanc) |
| PV1085 | Vaud-Valais | 6.7716 | 46.48041 | 15 | SUS | Chasselas |
| PV1090 | Vaud-Valais | 6.7716 | 46.48041 | 15 | SUS | Chasselas |
| PV1487 | Vaud-Valais | 6.66839 | 46.512305 | 16 | RES | Regent |
| PV1614 | Vaud-Valais | 6.66839 | 46.512305 | 16 | RES | Regent |
| PV1077 | Vaud-Valais | 6.25206 | 46.42414 | 17 | SUS | Gamay |
| PV1080 | Vaud-Valais | 6.25206 | 46.42414 | 17 | SUS | Gamay |
| PV1115 | Vaud-Valais | 6.23494 | 46.40631 | 18 | SUS | Chasselas |
| PV1566 | Vaud-Valais | 6.23097 | 46.39866 | 19 | RES | Bronner |
| PV1095 | Vaud-Valais | 6.23097 | 46.39866 | 19 | RES | Johanniter |
| PV1099 | Vaud-Valais | 6.23097 | 46.39866 | 19 | RES | Johanniter |
| PV1102 | Vaud-Valais | 6.23097 | 46.39866 | 19 | RES | Johanniter |
| PV1581 | Vaud-Valais | 6.23097 | 46.39866 | 19 | RES | Solaris |
| PV1598 | Vaud-Valais | 6.23097 | 46.39866 | 19 | RES | Solaris |
| PV1989 | Burgundy | 4.644971 | 45.956998 | 20 | SUS | Gamay |
| PV1993 | Burgundy | 4.644971 | 45.956998 | 20 | SUS | Gamay |
| PV1980 | Burgundy | 4.6509 | 45.972092 | 21 | SUS | Gamay |
| PV1202 | Burgundy | 4.73912 | 46.94757 | 22 | SUS | Chardonnay |
| PV1206 | Burgundy | 4.73912 | 46.94757 | 22 | SUS | Chardonnay |
| PV1395 | Burgundy | 4.73912 | 46.94757 | 22 | SUS | Chardonnay |
| PV1397 | Burgundy | 4.73912 | 46.94757 | 22 | SUS | Chardonnay |
| PV1399 | Burgundy | 4.73912 | 46.94757 | 22 | SUS | Chardonnay |

**Table S2.** Mixed linear models of the effects of inoculated host and source host (Bronner, Prior, Regent, other partially resistant varieties, *V. vinifera* cultivars) and their interaction (fixed effects) on quantitative pathogenicity traits in *P. viticola* (Table 2). Pathogen isolate nested in source host was entered as random in the model. A Moran’s I parametric test was performed on model residuals: no significant spatial autocorrelation was detected in any traits (*Z*-scores and *P*-values are reported).

| Source | Inoculated  host (H) | Source  host (S) | Interaction  (H x S) | | Moran’s I |
| --- | --- | --- | --- | --- | --- |
| **Main pathogenicity traits** | | | |  |  |
| Spore production | F_3,1278_=399.28  ***P*<0.0001** | F_4,98_=6.18  ***P* =0.0002** | F_12,1278_=4.76  ***P* <0.0001** | | -0.59  *P*=0.56 |
| Spore size | F_3,1278_=162.81  ***P* < 0.0001** | F_4,98_=4.61  ***P* =0.0019** | F_12,1278_=5.11  ***P* <0.0001** | | -1.450  *P*=0.13 |
| Latency period | F_3,1294_=58.24  ***P* < 0.0001** | F_4,98_=9.18  ***P* <0.0001** | F_12,1294_=8.58  ***P* <0.001** | | -1.13  *P*=0.26 |
| **Sporulation dynamics** | | | |  |  |
| *T*_50_ | F_3,1234_=156.35  ***P* <0.0001** | F_4,98_=2.12  *P*=0.08 | F_12,1234_=8.98  ***P* <0.0001** | | -1.309  *P*=0.19 |
| Sporulation rate | F_3,1234_=95.98  ***P* <0.0001** | F_4,98_=4.06  ***P* =0.0043** | F_12,1234_=2.11  ***P* =0.014** | | -0.99  *P*=0.32 |

^P < 0.05 statistics are indicated in bold.P=^

**Table S3.** Quantitative traits of *P. viticola* isolates from two origins (‘RES’ isolates sampled on partially resistant grapevine hosts and ‘SUS’ isolates sampled on susceptible *V. vinifera* cultivars) and used to inoculate Bronner, Prior, Regent and *V. vinifera* cv. Cabernet sauvignon. Sample size (*n*), mean (m), standard error (SE) and coefficient of variation (CV) of each trait are presented. Quantitative traits are defined in Table 2.

|  |  |  | Spore production (SPO) | | | Spore size | | | Latency period | | | *T*_50_ | | | Sporulation rate | | |
| --- | --- | --- | --- | --- | --- | --- | --- | --- | --- | --- | --- | --- | --- | --- | --- | --- | --- |
| Inoculated  host | Pathogen  origin | *n* | m | SE | CV | m | SE | CV | m | SE | CV | m | SE | CV | m | SE | CV |
| Bronner | RES | 46 | 86.50 | 9.01 | 69.85 | 11.50 | 0.13 | 7.42 | 3.29 | 0.06 | 12.34 | 3.31 | 0.05 | 10.63 | 35.62 | 3.78 | 69.53 |
|  | SUS | 47 | 50.35 | 3.68 | 50.10 | 11.80 | 0.10 | 6.05 | 3.49 | 0.08 | 15.25 | 3.21 | 0.04 | 9.01 | 31.93 | 3.78 | 77.69 |
| Prior | RES | 54 | 298.86 | 18.25 | 44.86 | 10.69 | 0.11 | 7.28 | 3.31 | 0.05 | 10.63 | 3.91 | 0.03 | 4.86 | 28.84 | 1.65 | 42.06 |
|  | SUS | 49 | 188.31 | 11.35 | 42.18 | 11.25 | 0.09 | 5.31 | 3.63 | 0.05 | 8.93 | 3.85 | 0.04 | 7.65 | 33.30 | 1.85 | 38.82 |
| Regent | RES | 53 | 359.21 | 21.56 | 43.69 | 10.85 | 0.11 | 7.17 | 3.03 | 0.02 | 4.76 | 3.35 | 0.04 | 9.73 | 28.62 | 2.12 | 53.91 |
|  | SUS | 49 | 274.79 | 12.85 | 32.73 | 11.50 | 0.10 | 5.81 | 3.03 | 0.02 | 4.04 | 3.71 | 0.04 | 7.52 | 21.46 | 1.78 | 58.01 |
| Cabernet  sauvignon | RES | 54 | 627.07 | 24.75 | 29.00 | 10.09 | 0.10 | 7.08 | 3.02 | 0.01 | 2.36 | 3.28 | 0.03 | 7.22 | 12.58 | 1.16 | 68.05 |
|  | SUS | 49 | 655.04 | 31.96 | 34.15 | 10.44 | 0.12 | 7.95 | 3.03 | 0.01 | 2.87 | 3.34 | 0.03 | 6.90 | 11.32 | 1.00 | 62.06 |

**Table S4.** Evolvability (*e_μ_*) and additive genetic variance (V_A_) of spore production (SPO) in *P. viticola* isolates from different origins (‘RES’ and ‘SUS’), for each inoculated resistant variety. The percent change over *t* generations with an evolvability of *e_μ_* and strength of selection *β_μ_* is (1 + *e_μ_ β_μ_*)*t* . We estimated *t*, the number of generations with *β_μ_* = 0.3, the median bias-corrected estimate of multivariate gradients from the meta-analysis of Hereford et al. (2004). SPO_SUS_ and SPO_RES_ for each resistant variety are indicated in Table S3.

|  | Average SPO (m) | V_A_ (SE) | *e_μ_* | Observed percent change in  spore production from SUS to RES  (SPO_SUS_ – SPO_RES_)/SPO_RES_ x 100 | *t* |
| --- | --- | --- | --- | --- | --- |
| Bronner | 68.4 | 778.44 (341) | 0.16% | 41.8 | 8 |
| Prior | 243.6 | 7056.7 (1834) | 0.12 % | 37 | 9 |
| Regent | 317 | 3720 (2727) | 0.04 % | 23.5 | 20 |

**Figure S1.** Map of *Plasmopara viticola* sampling, at 22 locations within five vine-growing regions: Burgundy, Alsace-Baden, Vaud-Valais, Tessino and Zurich. The number of geographic locations sampled and the number of isolates collected from partially resistant varieties (‘RES’) and *V. vinifera* susceptible cultivars (‘SUS’) is indicated for each region. Overall sample size was *n* = 54 and *n* = 49 isolates, for ‘RES’ and ‘SUS’ host of origin, respectively.

**Figure S2.** Specificity of *P. viticola* isolates for their source host of origin. None of the local isolates (black bars) was more aggressive (higher spore production) than other isolates, on any of the inoculated hosts (Table S2). Vertical bars represent mean values of spore production (number of sporangia / mm^2^) by *P. viticola* isolates from Bronner (‘BRO’), Prior (‘PRI’), Regent (‘REG’), other partially resistant varieties (‘OTH’) and *V. vinifera* cv. cultivars (‘SUS’) and used to inoculate Bronner, Prior, Regent (partially resistant varieties) and *V. vinifera* cv. Cabernet sauvignon. Black bars represent isolates collected from a focal variety and used to inoculate the same variety (local isolates). Different letters indicate significant differences among source hosts for each inoculated host (post-hoc least-squares mean differences). Sample sizes are: ‘BRO’: *n* = 9 on Bronner and *n* = 10 on Prior, Regent and Cabernet sauvignon; ‘PRI’: *n* = 10 on Bronner, Prior, Regent and Cabernet sauvignon; ‘REG’: *n* = 7 on Bronner and Regent, *n* = 8 on Prior and Cabernet sauvignon; OTH’: *n* = 20 on Bronner and *n* = 26 on Prior, Regent and Cabernet sauvignon and ‘SUS’: *n* = 47 on Bronner and *n* = 49 on Prior, Regent and Cabernet sauvignon.

**Figure S3.** Quantitative traits characterizing the sporulation dynamics of *P. viticola* isolates from two origins (‘RES’ isolates collected from partially resistant grapevine hosts, dark green solid line, n=54; and ‘SUS’ isolates collected from susceptible *V. vinifera* cultivars, light green dashed line, n=49). Isolates were used to inoculate Bronner, Prior, Regent and *V. vinifera* cv. Cabernet sauvignon (Cab. sauv.). Mean ±SE values of isolates by pathogen origin and frequency density plots are presented for each inoculated host on the left and right-hand side panels, respectively. (A) *T_50_*: time to 50% of final sporulation (5 dpi); (B): sporulation rate (slope at *T_50_*). See Table 2 for trait description and Table 3 for full model statistics. Post-hoc least-squares mean differences between pathogen origins (‘SUS’ and ‘RES’) on each inoculated host were determined and the significance of these differences is indicated as follows: *** *P*<0.0001.

**Figure S4**. Specificity of *P. viticola* isolates for resistant grapevine varieties considering traits related to sporulation dynamics. Mean (± SD) values for quantitative pathogenicity traits (A: *T*_50_; B: sporulation rate) are presented for each inoculated resistant host, for local and foreign isolates. Local isolates were collected from a focal resistant variety and used to inoculate the same variety (Bronner: *n* = 9; Prior: *n*=10; Regent: *n*=7), whereas foreign isolates were collected from a focal resistant variety and used to inoculate a different variety (Bronner: *n* = 37; Prior: *n*=44; Regent: *n*=46). See Table 5 for statistical analyses.
